# Intermittent light program impacts on reproductive performance, health and welfare of breeding hens

**DOI:** 10.5194/aab-66-315-2023

**Published:** 2023-11-14

**Authors:** Mohamed F. A. Farghly, Rashed A. Alhotan, Khalid M. Mahrose, Youssef A. Attia, Mostafa Abdelfattah, Mohammed Abougabal, Mossad Taboosha, Mohammed Ghonime, Mahmoud Shaaban, Caterina Losacco, Vincenzo Tufarelli

**Affiliations:** 1 Poultry Production Department, Agriculture College, Assiut University, Assiut, Egypt; 2 Department of Animal Production, College of Food and Agricultural Sciences, King Saud University, Riyadh, Saudi Arabia; 3 Animal and Poultry Production Department, Faculty of Technology and Development, Zagazig University, Zagazig, Egypt; 4 Animal and Poultry Production Department, Faculty of Agriculture, Damanhour University, Damanhour 22516, Egypt; 5 Animal Production Department, Agriculture College, Al-Azher University, Cairo, Egypt; 6 Animal and Poultry Production Department, Agriculture and Natural Resources College, Aswan University, Aswan, Egypt; 7 Poultry Production Department, Faculty of Agriculture, New Valley University, New Valley, Egypt; 8 Department of Precision and Regenerative Medicine and Jonian Area, Section of Veterinary Science and Animal Production, University of Bari Aldo Moro, Valenzano, Bari, Italy

## Abstract

The lighting regime significantly impacts poultry production, reproductive performance, health and welfare. This study aimed to test the effect of the intermittent light (IL) regime on reproductive organs and hormones, semen quality, and behavioral performance. Thus, 270 Rhode Island Red hens aged 20 weeks were distributed among three groups of six replicates and 15 birds each, housed in floor pens. The first group was used as a control (C) and was exposed to constant light for 16 h d ${}^{-1}$ , while birds in other groups were exposed to IL for 20 min h ${}^{-1}$ (T20) and 40 min h ${}^{-1}$ (T40) during the 16 h light period. The outcomes were that follicle number was higher for T20 compared to T40 but not the controls, while T40 is different from T20 but not the controls. The same is true for testosterone. The sperm concentration is lower for T40 compared to the controls, with no difference between T20 and the controls. Body temperature was not different among groups. Conversely, differences were not noticed for leg alterations; plumage conditions; and relative ovary, oviduct, and/or testes weight and hatchability. Thus, the IL T20 program should be further investigated as a lighting regimen for managing Rhode Island Red laying hens for stimulating follicle number and testosterone without negatively impacting the physiological response and health traits. From a practical point of view, the IL schedule of 20 min h ${}^{-1}$ during 20–36 weeks of age can be economically viable due to saving 66 % of the light cost.

## 1 Introduction

Although the egg production industry has advanced immensely throughout the latter years because of the introduction of high-egg-producing strains, numerous management aspects still need to be adopted to obtain ideal performance (Farghly et al., 2019a). Light management is vital among the diverse factors of management (Wang et al., 2015; Abo Ghanima et al., 2020; Farghly et al., 2020). The light regime is effective in increasing the reproductive performance of poultry (Jácome et al., 2014). Light is the element responsible for the release of hormones in the reproduction of poultry (Patel et al., 2016). There has been much emphasis on the influences of lighting regimes on sexual maturity, egg production, behavior, physiology, welfare, health, fertility and hatchability (Perićet al., 2005; Mohammed et al., 2010; Yang et al., 2015; Mohammed, 2016). With the knowledge of melatonin being synthesized in the retina, the importance of light prevails over other environmental issues (El-Badry et al., 2015; Demirbas and Kubanc, 2018). Recently, an IL system for broilers could effectively enhance their physiological status and growth performance under heat-stress conditions (Alaqilet al., 2022). On the other hand, light regimes affected the duration and frequency of circadian behavior of laying hens, and continuous light was more beneficial to reproductive development than the IL during 22–30 weeks of age (Geng et al., 2022).

Feed consumption should be carefully considered in the egg production industry (Farghly et al., 2019a). Feed intake could be reduced when applying IL regimes (Patel et al., 2016), enhancing feed efficiency (Bahloul et al., 2014). Light flashes could lower expenses by lessening electricity costs (Farghly et al., 2016). Several investigations on the optimal use of electric power in poultry production are significant, as the conservation of energy supplies is vital for the sustainable development of egg production (Yuri et al., 2016). Continuous lighting programs may instigate numerous welfare-associated issues (Bayram et al., 2010; El Sabry et al., 2019; Mohammed, 2019), while suitable photoperiod programs (length and intensity) have a beneficial effect on the stress and welfare of broilers, including health of eyes (Fidan et al., 2017) and physical activity and natural behaviors, which may enhance footpad status and health of legs of broilers (Kang et al., 2023). The light scheme was extensively beneficial for boosting the reproductive performance of laying hens (Mohammed, 2016). The plan and length of the lighting pattern let the fowl display a circadian pattern of the events of egg formation and oviposition (Farghly et al., 2019a). The cyclical sexual efficacy and behavior of geese are motivated by short lighting periods (Huang et al., 2008).

Recent approaches to understand the effect of the light program and the financial deficiencies in more depth were directed to an altered awareness in advancing applications in chicken management by exposing birds to a photoperiod which comprises light flashes (Farghly et al., 2015, 2019; Geng et al., 2022). It is not recognized whether light flashes as bio-intermittent light create variation compared to continuous light stimulation, which may be valuable to know.

Therefore, this study was to examine the relationship between light flashes for a 20 min (light) and 40 min (dark) light period and light flashes for a 40 min (light) and 20 min (dark) light period for 16 h d ${}^{-1}$ compared to persistent light for 16 h d ${}^{-1}$ and reproductive organs and hormones, semen quality, and behavioral performance of Rhode Island Red hens and cocks.

## 2 Materials and methods

The research was conducted at the Research Poultry Farm, Poultry Production Department, Faculty of Agriculture, Assiut University, Egypt.

### 2.1 Experimental animals

A total of 270, 20-week-old Rhode Island Red hens and cocks (average body weight was 1490 g for females and 1640 g for males) were separated into three groups (six replicates of 15 birds each, 12 females and 3 males) and housed in floor pens ( 1×2 m) in an open-sided house.

### 2.2 Experimental design, lighting programs and birds' management

The house and management conditions were previously described by Farghly et al. (2019a) but are given briefly as follows: the first group was considered the control (C) and was subjected to persistent light for 16 h light per day, while birds in the other two groups were exposed to light flashes/intermittent light for a 20 min (light) : 40 min (dark) light period (T20) and light flashes for a 40 min (light) : 20 min (dark) light period (T40), correspondingly, during the 16 h light period. All natural light sources were removed with thick cotton dark draperies and shutdown flexible draperies, which wholly eliminated any supply of normal light. Light intensity was determined at the pen's center and ranged from 15 to 20 lx by operating incandescent bulbs positioned 1.5 m from the ground. Light flashes were described as flashing illuminations with appropriate intensity at the bird's level, produced by a flasher device encompassing a timer and dimmer to validate the flashed lighting phase and intensity. The light schedule started at 06:00 and ended at 22:00. The intermittent lighting is measured by the frequency of light and dark pulses per minute. The experimental birds were maintained under temperature conditions of 24–26 ${}^{\circ }$ C and relative humidity of 55 %–65 % during the testing period (16 weeks). Feed and water were freely accessible, and all the other environmental and managerial conditions were the same during the investigational phase (20–36 weeks of age). The calculation and the composition analysis of the diets are shown in Table 1, as explained earlier (Farghly et al., 2019a).

**Table 1:** Composition and calculated analysis of experimental diet.

Ingredients	(%)
Yellow corn	69.5
Soybean meal (44 % CP)	15.0
Limestone	7.0
Concentrate ${}^{*}$	8.0
Bone meal	0.4
Salt	0.1
Calculated analysis	
Crude protein (%)	17.4
Metabolizable energy (kcal kg ${}^{-1}$ ) ${}^{**}$	2867
Calcium (%)	3.10
Available phosphorus (%)	0.37

${}^{*}$ Layer concentrate: crude protein 51.0 %, lysine 3.3 %, crude fiber 2.0 %, calcium 8.0 %, crude fat 6.4 %, available phosphorus 3.0 %, methionine 1.7 %, salt, 3.2 %, methionine + cystine 2.25 %, metabolizable energy 2400 kcal per diet. Each kilogram of layer concentrate contains the following levels of vitamins and minerals: vit. A; 10000 IU folic acid, 10 mg vit. E, 100 mg biotin, 500 mg vit. D3, 2500 IU chorine chloride, 5000 mg vit. K, 25 mg iron, 400 mg vit. B1, 100 mg zinc, 560 mg vit. B2, 40 mg copper, 5 mg vit. B6, 15 mg iodine, 3 mg vit. B12, 200 mg selenium, 1 mg pantothenic acid, 100 mg manganese, 620 mg niacin, 400 mg antioxidant 75 mg. ${}^{**}$ NRC (1994).

### 2.3 Data collection

Blood samples ( n=6 per treatment) were gathered at 10:00 from hens with eggshells in the uterus. The blood was collected in heparinized tubes and centrifuged at 3000 rpm for 15 min, and plasma gained was kept at -20 ${}^{\circ }$ C pending investigation Research Park, Cairo University, Giza, Egypt). Testosterone, estradiol-17b (E2), progesterone, luteinizing hormone (LH) and follicle-stimulating hormone (FSH) were assessed via a radioimmunoassay (Schanbacher and D'Occhio, 1982; Onagbesan et al., 2006). The hormones intraassay CV <5 %–10 %, and the interassay CV <9 –12 with a recovery range of 77 %–105 % depending on the hormone.

For assessing leg problems (hock discoloration and/or footpad dermatitis), the scores extended from 1 (no hock discoloration or footpad dermatitis) to 5 (whole coverage of red hock discoloration or total footpad involvement in footpad dermatitis), as indicated earlier (Farghly et al., 2019b). Body temperature ( ${}^{\circ }$ C) was measured biweekly in the morning (06:00 to 07:00), using a thermometer introduced into the rectum for 2 min at a depth of 2 cm. Plumage condition scores were determined at ages of 24 and 36 weeks. Three regions of the body were evaluated (head, neck and back) on a scale from 1 (fully feathered) to 5 (featherless). Mortalities were recorded daily, and the mortality rate was expressed as a percentage of the total birds at placement. Tonic immobility is a traditional measure of fearfulness in poultry (Campo et al., 2002). Tonic immobility was induced by placing the bird on its back and restraining it for 15 s. If the bird remained immobile for 15 s after the experimenter removed their hands, a stopwatch was started to record latencies (s) until the bird righted.

At 16 weeks of age, all males were independently tested at periods of 1 to 2 weeks for the commencement of semen production by physical massage. Commencement of mature semen production was appraised by eye and scored (Farghly et al., 2017) on a scale of 6 to 1, as follows: where 6 indicates cloacal with a shrunken exit; 5 indicates extrusion of the rudimentary penis, without semen production; 4 indicates the production of seminal fluid; 3 indicates the production of yellow semen; 2 shows some indication of white semen; and 1 indicates the production of white semen. A score of 1 was specified to characterize the success of abundant semen production of the male. At this time, body weight and age were obtained for the bird. Semen was gathered into a graduated gathering tube to record the volume per ejaculate with a precision of 0.05 mL. After collection, tubes were preserved at 38–40 ${}^{\circ }$ C in a thermos flask. Sperm concentration (millions per milliliter) was determined using a hemocytometer (Thoma) (Salisbury et al., 1985). At 36 weeks, six females and three males per group were taken and slaughtered in the morning (07:00 to 08:00).

At the end of the experiment, the genital organs as a weight relative to live body weight (testes in males and ovary, oviduct, oviduct length and follicle number in females) were determined, after slaughtering, when birds were sexually mature, measured by 50 % of egg production for female and semen production for male (Farghly et al., 2017). A total of 126 eggs per group (21 eggs per replicate) were randomly chosen (the incubation process was carried out three times during 35–36 weeks of age, as seven eggs per replicate each time) and were incubated under the same conditions (37.5 ${}^{\circ }$ C and 60 % RH during the first 18 d and then 37 ${}^{\circ }$ C and 75 % relative humidity during the last 3 d). Eggs were candled on day 7 of incubation to determine fertile eggs. Fertility and hatchability percentages were calculated as follows: fertility (%) = (number of fertile eggs/total eggs set) ×100 , and hatchability on fertile egg basis (%) = (viable hatched chicks/number of fertile eggs) ×100 .

### 2.4 Statistical analysis

Data gathered were subjected to analysis of variance (ANOVA) by utilizing the general linear model method of SAS software (SAS, 2019). Variances among averages of the different groups were identified (Duncan, 1955). The pen was the experimental unit. After analysis, the percentages were transformed to Arcsin values and then re-transformed to the original values. Logarithmic transformation was used before analysis. Significance was set at the 5 % level. The next equation was depleted for examination of variance: $Y_{ij}=µ+S_{i}+e_{ij}$ , where $Y_{ij}$ is the observation, µ is the overall mean, $S_{i}$ is the treatment impact and e ${}_{ij}$ is the investigational errors. The Tukey post hoc test examined mean differences among treatments.

**Table 2:** Effect of light flash program on genital organs and age at sexual maturity. SEM: standard error of means.

Traits	Treatments			SEM	P value
	C	T20	T40		
Genital organs					
Testes, %	1.60	1.57	1.51	0.36	0.3265
Ovary, %	3.21	3.66	3.48	0.49	0.2617
Oviduct, %	2.63	2.58	2.54	0.38	0.6942
Oviduct length, cm* 58.9	60.1	57.8	2.33	0.4826	
Follicle number	6.36 ${}^{\text{a, b}}$	7.00 ${}^{\text{a}}$	5.03 ${}^{\text{b}}$	0.62	0.0243
Age at sexual maturity (days)					
For females (at 50 % egg production)	156.7	155.7	158.7	2.21	0.7180
For males (semen production)	161.0	161.2	162.2	1.97	0.5487

${}^{\text{a, b}}$ Means within a row followed by different superscripts are significantly different ( P≤0.05 ). C: birds were exposed to common light. T20 and T40: birds were exposed to light flashes for 20 and 40 min per hour of light, respectively. Testes, ovary and oviduct (%) show the weight relative to 100 g body weight.

## 3 Results

### 3.1 Genital organs and sexual maturity

Laying hens kept under T20 produced the highest ( P<0.05 ) number of follicles in comparison with the other groups (Table 2). However, the changes in the different traits of genital organs and sexual maturity did not reach the significance level among the studied treatments (Table 2).

**Table 3:** Effect of light flash program on blood hormones.

Traits	Treatments			SEM	P value
	C	T20	T40		
Testosterone (ng mL ${}^{-1}$ )	3.10 ${}^{\text{a,b}}$	3.26 ${}^{\text{a}}$	2.62 ${}^{\text{b}}$	0.17	0.0452
Estradiol-17b, E2 (pg mL ${}^{-1}$ )	149.6	155.2	147.6	4.28	0.7426
Progesterone, P4 (ng mL ${}^{-1}$ )	0.33	0.42	0.39	0.03	0.2569
Luteinizing hormone, (ng mL ${}^{-1}$ )	3.06	2.88	2.81	0.63	0.4355
Follicle-stimulating hormone, (ng mL ${}^{-1}$ )	4.68	4.92	4.66	0.75	0.4682

${}^{\text{a, b}}$ Means within a row followed by different superscripts are significantly different ( P≤0.05 ). C: birds were exposed to common light. T20 and T40: birds were exposed to light flashes for 20 and 40 min per hour of light, respectively.

### 3.2 Blood hormones

Results presented in Table 3 indicate that males of the T20 group and those of the control had the highest ( P<0.05 ) testosterone concentration compared with those of T40. Non-significant differences were observed for the other blood hormones of treated hens and those of the control (Table 3).

**Table 4:** Effect of light flash program on physiological and health aspects.

Traits	Treatments			SEM	P value
	C	T20	T40		
Leg problems	2.00	1.88	2.16	0.71	0.8123
Body temperature ( ${}^{\circ }$ C)	41.22	40.15	41.6	0.24	0.0542
Plumage conditions	2.07	1.60	1.91	0.68	0.2844
Tonic immobility	1.78 ${}^{\text{a, b}}$	1.67 ${}^{\text{b}}$	1.82 ${}^{\text{a}}$	0.084	0.0063
Mortality rate (%)	3.33	2.22	4.44	3.44	0.5466

${}^{\text{a, b}}$ Means within a row followed by different superscripts are significantly different ( P≤0.054 ). C: birds were exposed to common light. T20 and T40: birds were exposed to light flashes for 20 and 40 min per hour of light, respectively.

### 3.3 Physiological and health aspects

It could be said from the data displayed in Table 4 that the group of hens reared under T20 had significantly ( P<0.05 ) lower tonic immobility than the other groups. We did not find statistical support ( P>0.05 ) for differences in body temperature, leg problems (%), plumage conditions and mortality rate (%) among the birds of the different experimental groups.

**Table 5:** Effect of light flash program on semen quality traits, fertility and hatchability.

Traits	Treatments			SEM	P value
	C	T20	T40		
Semen quality					
Reaction time (s)	28.65	29.21	39.89	2.11	0.2514
Semen color (1–3)	1.31	1.37	1.33	0.35	0.4161
Semen pH	7.00	7.20	6.91	0.66	0.5685
Semen volume (mL)	0.42	0.41	0.39	0.09	0.4172
Sperm-cell concentration (10 ${}^{9}$ mL)	4.89 ${}^{\text{a}}$	4.56 ${}^{\text{a, b}}$	4.21 ${}^{\text{b}}$	0.24	0.0003
Incubation traits					
Fertility, %	91.42 ${}^{\text{a}}$	91.72 ${}^{\text{a}}$	88.11 ${}^{\text{b}}$	2.45	0.0280
Hatchability, %	75.42	75.22	73.81	2.94	0.6512

${}^{\text{a, b}}$ Means within a row followed by different superscripts are significantly different ( P≤0.05 ). C: birds were exposed to common light. T20 and T40: birds were exposed to light flashes for 20 and 40 min per hour of light, respectively.

### 3.4 Semen quality, fertility and hatchability of eggs

Data presented in Table 5 clearly show significant differences ( P<0.05 ) in sperm-cell concentration and fertility (%) among the experimental groups. Sperm-cell concentration was the highest in males of the control group, followed by those of T20 and then T40, with the difference between the control and T40 being significant ( P<0.05 ). Males of T20 and control groups had higher fertility than those reared under T40. However, there were non-significant differences in the other traits of semen quality and hatchability %.

## 4 Discussion

The current findings suggested that IL, as a lighting scheme of 20 min or 40 min during 20–36 weeks of age, has no detrimental effects on the reproductive and behavioral performance of Rhode Island Red laying hens. Reproductivity in laying hens depends on an endogenous mechanism closely related to external factors, and the number of follicles in the hen determines the laying rate (Farghly et al., 2019a; Hao et al., 2020). The synchronization of these factors is called the circadian rhythm and allows ovulation to occur regularly during lay. Layers use circadian rhythms to perceive the day, and they are most sensitive to light (11 and 15 h) after the light is initiated (Jácome et al., 2014; Geng et al., 2022).

Understanding light and dark cycles enables the bird to distinguish a subjective day and controls ovulation and oviposition (Perry and Lewis, 1993). Keeping growing hens under an IL program (2 h light + 1 h dark) ×4+12 h dark until 17th week of age) resulted in an increasing number of follicles at the 18th week of age and development of the ovaries and oviducts (Perićet al., 2005).

In the present study, ovarian weight tended to be lower than the extended lighting periods used in the current research. This is supported by the findings of Chen et al. (2007). Moreover, our results are in line with those obtained by Perić et al. (2005), who found that hens at first egg lay had, on average, 5.5–6.5 mature large ovarian follicles.

Light stimulation encourages pituitary gland anterior lobe activity, which organizes the excretion of FSH (Farghly et al., 2017). This accelerates the growth of ovarian follicles, which mature to yield eggs (Hao et al., 2020). There is an association between melatonin and follicle growth, gonadal size, or sexual development (Çalişlaret al., 2018; Hao et al., 2020). Furthermore, it has been suggested that melatonin may act as a chief photoperiodic indicator to stimulate the reproductive alignment of birds (Jácome et al., 2014; Talpur et al., 2018). Therefore, light limitation protocols are currently applied to reproductive activity (Farghly et al., 2019a). Hao et al. (2020) observed that melatonin administration to aged laying hens improved the numbers of medium and small white follicles.

In the current treatments, increasing periods of darkness may support increased melatonin secretion, thus enhancing follicle number. Farghly (2014) found differences in the age at sexual maturity (females and males), fertility, genital organs (ovary, testes percentages and follicle number) and semen quality (semen volume and sperm-cell concentration) among the experimental groups exposed to light flashes. On the other hand, Farghly (2014) and Farghly et al. (2017) found non-significant differences in oviduct length and percentage, semen color and pH, reaction time, and hatchability percentage due to IL.

It was found that IL increased follicle number, and the T20 group had the greatest number and testosterone concentration compared to the T40 scheme but comparable to the continuous control. The findings showed that IL did not influence physiological response, health and behavior, suggesting that IL might be an alternative to constant light for managing Rhode Island Red hens.

In the literature, it is shown that significantly prolonged photoperiods can limit reproductive maturity in laying chickens (Chen et al., 2007). Photo-stimulation is associated with elevated LH and FSH excretion from the anterior pituitary gland, which, in sequence, supports testicular development and Leydig cell multiplication (Henare et al., 2011). Chen et al. (2007) found that the photoperiod had a limited impact on ovarian follicle formation, whereas the photoperiod controlled oviduct and ovary development. Ovarian weight appeared lower, and lipid stores were raised relative to the extended photoperiod groups.

The current results align with those reported by Farghly et al. (2017), who found no non-significant changes in measured blood hormones, except for estradiol, in chickens exposed to light flashes. Farghly et al. (2017) attributed the variations in blood hormones to the absence of the physiological stress or the negative effect after exposing birds to short-flashed-light treatments. Hao et al. (2020) indicated that melatonin administration to aged laying hens (70 weeks old) enhanced the reproductive hormones estradiol and LH in the plasma. Melatonin secretion increases in darkness (Talpur et al., 2018). In the current work, IL in T20 had the greatest release of sexual hormones (E2, P4 and FSH) and was significant only for testosterone. On the other hand, plasma LH concentration of laying hens was superior in hens exposed to 12 h light : 12 h dark during 16–26 weeks of age, while birds reared under 8 h light : 16 h dark had greater LH concentration through 38–43 weeks of age, but no differences were recorded between 30–34 weeks of age (Classen et al., 2004). The same researchers demonstrated that LH is identified as the primary hormone regulating reproduction in poultry as it encourages gonadal steroid formation and/or secretion and synchronizes the sex steroid hormones and ovulation.

The present findings emphasize the impacts of IL on plasma testosterone concentration, showing that T20 enhanced its secretion. The variations in the excretion of sexual hormones are inducible by artificial lighting programs (Shi et al., 2007). Testosterone is considered an indicator of male fertility, and spermatogenesis depends on testosterone secretion (Tyler and Gous, 2008). Also, studies on turkey males subjected to continuous or IL routines showed slight changes in LH and testosterone secretion (Bacon et al., 2000). In addition, Lewis et al. (2009) found that photoperiod significantly affected testicular weight. However, Noirault et al. (2006) found that males in different photoperiod groups had similar reproductive characteristics. The same investigators stated that plasma LH and testosterone concentrations were poor indicators of testis development and semen production, irrespective of age and photoperiod. Intermittent lighting supports normal semen production in turkeys (Bacon et al., 1994). In addition, Tyler et al. (2011) observed no photoperiodic effect on sperm concentration and output. In accordance with the present results, immature turkey males initially exposed to a short (6 h light : 18 h dark) and then a longer (16 L : 8 D) photoperiod had markedly higher plasma LH and testosterone and consequently testis weight than other lighting treatments but not semen output (Yang et al., 1998). Furthermore, reducing daily photoperiods from long to short reduced LH and testosterone concentrations (Yang et al., 2017).

The impact of light scheme on semen quality was reported in the literature by Farghly et al. (2017), who stated that exposing male Sharkasi chicken to 10–30 min per hour of IL resulted in the highest sperm-cell concentration and fertility when compared with longer periods of IL or the control. Spermatogenesis depends on testosterone, which can also be measured as an indicator of fertility (Tyler and Gous, 2008). Noirault et al. (2006) endorsed that mature turkey males can be moved and sustained in semen production below lighting schedules of 9.5–10.5 h light : 13.5–14.5 h dark. Similar results were also reported by Farghly (2014). However, Farghly et al. (2016) indicated that birds subjected to a continuous light system gave higher semen quality characters and enhanced fertility and hatchability percentages than those in the IL scheme, excluding semen pH values.In addition, Wang et al. (2015) concluded that fertility was greater in pigeons exposed to 15 h light : 9 h dark than those exposed to full natural light. On the other hand, fertility and hatchability were unchanged by the adolescent or breeder light schedule (Onbaşılar et al., 2007).

Behavior is an excellent index for estimating the welfare of laying hens (Huber-Eicher et al., 2013). The present work observed that tonic immobility was lower in T20 than in the T40 group. Tonic immobility could influence exact extra welfare and production-associated characteristics, with possible production charges connected with tonic immobility behavior in poultry production (Fogelholm et al., 2019). The time consumed in tonic immobility and the number of inductions required per immobility test have been associated with behavioral and stress reactions (Bayram et al., 2010; Fogelholm et al., 2019). Mohammed (2016) concluded that the variation in the lighting period of layers is related to differences in behavior. In addition, Das and Lacin (2014) showed that the varied photoperiods exhibited no significant influence on tonic immobility or tibial dyschondroplasia estimates.

An increased fear response, as indicated by a longer tonic immobility for hens housed under 23 h light : 1 h dark ( 236±32 s) compared to 14 h light : 10 h dark ( 137±32 s), lead the authors to suggest that continuous lighting programs can result in passive outcomes that decrease welfare in egg-laying hens (Farghly et al., 2017). In Japanese quail, Demirbas and Kubanc (2018) stated that increasing darkness from 17 to 21 h d ${}^{-1}$ results in a similar degree of playing behavior. Bayram and Özkan (2010) observed that broilers kept in continuous light had experienced a superior fear reaction than birds reared under 12 h light : 12 h dark or 16 h light : 8 h dark photoperiods. Farghly et al. (2017) attributed the low body temperature values of hens exposed to IL to the short lighting programs (10–30 light flashes per minute). Comparable results were also confirmed by El-Badry et al. (2015), who used an IL regime of 4 h light L : 2 h dark, providing a total of 16 h light and 8 h dark for Muscovy ducks.

The current light treatments had no effects on mortality (%), which was in line with the results of Bahloul et al. (2014), and found non-significant variances in physiological and health aspects (leg problems, plumage conditions and mortality rate) in chickens exposed to light flashes.

Our outcomes related to mortality (%) were previously confirmed by Bahloul et al. (2014), who demonstrated that IL programs did not alter mortality (%) and thus welfare. Birds kept under extended periods of darkness are reported to be healthier than their counterparts under elongated daylight (Farghly, 2014). The IL schedule enhanced immunity by augmenting both humoral and cell-mediated reactions, which was the main issue in decreasing the mortality rate (Farghly and Makled, 2015).

The photoperiodic schedule can influence bird activity, which impacts exercise and accelerates bone density, thus increasing leg strength (Yang et al., 2015). The improved movement may increase bone strength growth (Buyse et al., 1996). Several weaknesses are related to constant lighting schemes. Hens are less dynamic, and leg complaints are more abundant and prevalent (Mohammed, 2019). Comparable conclusions were described by Farghly (2014), Yang et al. (2015) and Farghly et al. (2017), all of whom revealed that IL was not associated with leg problems. However, Tuleun et al. (2010) established that continuous lighting augmented the occurrence of leg abnormalities. Farghly and Makled (2015) found that IL considerably improved survival and reduced leg problems. The non-appearance of photoperiodic influences on mortality rate is in consonance with former outcomes (Ciacciariello and Gous, 2005). In previous studies, biomittent schedules were found to boost livability and reduce metabolic syndromes such as ascites, which are accompanied by certain illnesses (Onbasilar et al., 2007). Contrarily, less mortality has also been observed in broilers kept under IL compared to those with longer photoperiods (Lewis et al., 2009).

The numerical increase in sperm-cell concentration in the group of birds reared under T20 led to higher fertility than T40. Previous investigations indicated that decreasing lighting stimulated semen production and sperm-cell concentration (Shi et al., 2007).

## 5 Conclusions

Based on follicle number and testosterone concentration results, light flashes scheduled as biomittent light might be an economical alternative to continuous light for managing Rhode Island Red hens without harmfully influencing the physiological response, healthy traits, behavior and welfare. Finally, a light flash schedule of a 20 min : 40 min light period during 20–36 weeks of age is suggested for further experiments to confirm the economic and production benefits for the farming of breeding hens.

## Data Availability

Data supporting the findings of this study are available on request from the corresponding author. The data are not publicly available due to privacy or ethical restrictions.
